# Real-Time Split-Dose PET/CT-Guided Ablation Improves Colorectal Liver Metastasis Detection and Ablation Zone Margin Assessments without the Need for Repeated Contrast Injection

**DOI:** 10.3390/cancers14246253

**Published:** 2022-12-19

**Authors:** Mahdi Zirakchian Zadeh, Randy Yeh, Henry S. Kunin, Assen S. Kirov, Elena N. Petre, Mithat Gönen, Mikhail Silk, Francois H. Cornelis, Kevin C. Soares, Etay Ziv, Stephen B. Solomon, Vlasios S. Sotirchos, Constantinos T. Sofocleous

**Affiliations:** 1 Interventional Radiology/Oncology Service, Department of Radiology, Memorial Sloan Kettering Cancer Center, New York, NY 10065, USA; 2 Molecular Imaging and Therapy Service, Department of Radiology, Memorial Sloan Kettering Cancer Center, New York, NY 10065, USA; 3 Department of Medical Physics, Memorial Sloan Kettering Cancer Center, New York, NY 10065, USA; 4 Biostatistics Service, Memorial Sloan Kettering Cancer Center, New York, NY 10065, USA; 5 Department of Surgery, Memorial Sloan Kettering Cancer Center, New York, NY 10065, USA

**Keywords:** colorectal liver metastasis, tumor ablation, PET/CT, tumor detection rate, ablation margins, ablation zone assessment

## Abstract

**Simple Summary:**

This study showed that adding PET to CT-guided ablation of CLMs can improve tumor detection for targeting, allowing for repeated tumor visualization for monitoring during ablation and assessing the ablation zone and margins after treatment completion while eliminating the need for repeated contrast administration before and during the ablation.

**Abstract:**

Background: Real-time split-dose PET can identify the targeted colorectal liver metastasis (CLM) and eliminate the need for repeated contrast administration before and during thermal ablation (TA). This study aimed to assess the added value of pre-ablation real-time split-dose PET when combined with non-contract CT in the detection of CLM for ablation and the evaluation of the ablation zone and margins. Methods: A total of 190 CLMs/125 participants from two IRB-approved prospective clinical trials using PET/CT-guided TA were analyzed. Based on detection on pre-TA imaging, CLMs were categorized as detectable, non-detectable, and of poor conspicuity on CT alone, and detectable, non-detectable, and low FDG-avidity on PET/CT after the initial dose. Ablation margins around the targeted CLM were evaluated using a 3D volumetric approach. Results: We found that 129/190 (67.9%) CLMs were detectable on CT alone, and 61/190 CLMs (32.1%) were undetectable or of poor conspicuity, not allowing accurate depiction and targeting by CT alone. Thus, the theoretical 5- and 10-mm margins could not be defined in these tumors (32.1%) using CT alone. When TA intraprocedural PET/CT images are obtained and inspected (fused PET/CT), only 4 CLM (2.1%) remained undetectable or had a low FDG avidity. Conclusions: The addition of PET to non-contrast CT improved CLM detection for ablation targeting, margin assessments, and continuous depiction of the FDG avid CLMs during the ablation without the need for multiple intravenous contrast injections pre- and intra-procedurally.

## 1. Introduction

Local cure of colorectal cancer (CRC) liver metastasis (CLM) is challenging [1]. Up to 20% of CRC patients have synchronous, and up to 50% develop metachronous CLM within 3 years following resection of the primary [2]. Metastasectomy used as a local cure for CLM can improve survival in selected patients [3]. However, only 20–25% of patients with CLMs are candidates for resection [3]. Percutaneous ablative techniques are used to treat selected small CLMs either alone or in combination with resection, provided that all visible CLMs can be eradicated [4]. In patients with small liver volume disease that can be ablated with appropriate margins, local tumor control and patient survival after percutaneous thermal ablation (TA) (radiofrequency or microwave) is comparable to that of hepatectomy (up to 55% at 5 years) [5]. Despite the potential benefits, earlier reports of high local tumor progression (LTP) rates have limited the widespread use of TA to treat CLM [6]. Prior studies have consistently shown that the ablation margin is an independent predictor of LTP following ablation of CLM [6,7,8,9,10,11,12,13,14,15,16]. Because the majority of intrahepatic micro metastases are detected within 10 mm of the CLM’s border [6,7,8,9,10,11,12,13,14,15,17], an ablation zone (AZ) that extends beyond the tumor’s borders with over 10 mm of minimum ablation margins (MM) is ideal; whereas a 5 mm margin is regarded as the absolute minimum requirement when ablation is offered with local curative intent [6,10,14]. The precise characterization of the targeted tumor and its contours is the first critical step for the assessment of AZ and MM. The selection of a guidance system is influenced by tumor visibility, clinician preferences, and the availability of specialized equipment [18]. Ultrasound is probably the most extensively utilized technology, however, it is subject to poor tumor detection due to overlaying structures, a low tumor echogenicity gradient, and gas generation that prevents the accurate depiction of the AZ [18]. A three-dimensional image of the targeted tumor, surrounding structures, electrodes, and tissue changes is possible with conventional computed tomography (CT) fluoroscopy [18]. Despite these advantages, high radiation exposure, limited angulation possibilities during electrode insertion, short contrast-enhanced imaging time frame, and suboptimal visualization of intrahepatic vessels and bile ducts due to limited intravenous contrast agent administration pose significant drawbacks [18]. In a recent study, it was shown that compared to conventional CT fluoroscopy, transcatheter CT hepatic arteriography-guided ablation was associated with improved local disease control and superior liver tumor progression-free survival (LTPFS) [18]. The authors concluded that CT hepatic arteriography is a safe and effective alternative to CT fluoroscopy since it minimizes the number of repeat ablations required while posing no additional risk or compromising life [18].

Positron emission tomography (PET)/CT-guided ablation has also shown promise. Combining detailed anatomical information obtained by CT with the metabolic information from FDG-PET (PET/CT) during ablation offers advantages in tumor detection and contour definition, addressing the limitations of anatomic imaging alone [19]. PET avid tumors are visible on fluorodeoxyglucose (FDG) PET scans even when their echogenicity, attenuation, or signal intensity do not allow their detection by ultrasound, CT, or magnetic resonance imaging (MRI) [20]. Real-time PET/CT-guided ablation can eliminate the need for several contrast injections for targeting CLM and combines the anatomical details obtained from non-contrast CT and metabolic data obtained from PET. In addition, real-time PET/CT has the advantage of a continuous depiction of the FDG avid tumor during the procedure and allows for repeated PET image acquisition and tumor detection, an important feature, especially in case of tumor position change (patient movement or intraprocedural manipulations such as hydrodissection). The FDG dose for real-time PET is injected in two different aliquots; the first aliquot represents one-third of the diagnostic dose (4 millicurie (mCi) (148 megabecquerel (MBq)) and is used for the initial tumor targeting. The second aliquot – for treatment efficacy, is dose-equivalent to two-thirds of the diagnostic dose and is administered upon completion of the ablation to assess the treated tumor and the AZ. This technique is called split-dose [20]. 

This study aimed to assess the added value of pre-ablation real-time split-dose PET when combined with non-contract CT in the detection of CLM for ablation and the evaluation of the ablation zone and margins.

## 2. Materials and Methods 

### Study Design and Participants

**Figure 1:**
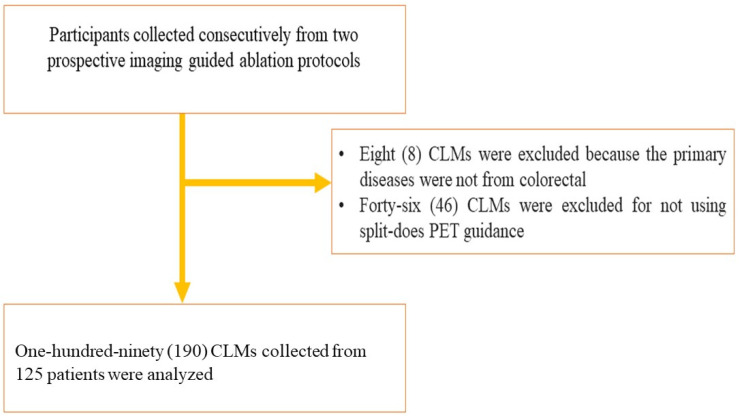
Study Flowchart.

This study is an analysis of two prospective institutional review board (IRB) approved/National Cancer Institute/National Institute of Health (NCI/NIH) supported studies performed in a single tertiary cancer center from October 2010 to May 2021 (Figure 1). ClinicalTrials.gov identifier (NCT) numbers are NCT04143516 and NCT01494324. The IRB protocol numbers are 19-332 and 09-122; corresponding NIH/NCI grants are RO1 CA 240569-01 and R21 CA131763-01A, respectively. The studies were conducted in accordance with the Health Insurance Portability and Accountability Act of 1996 (HIPAA) compliance for U.S. studies. Participant/tumor characteristics are summarized in Table 1. The study population includes participants with small CLMs (up to 5 cm) treated with TA according to institutional and interventional radiology service guidelines with the addition of PET/CT guidance using the split-dose technique, as previously described [20]. Participants who met institutional criteria for treatment with ablation, with no or limited and controlled/stable extrahepatic disease, were eligible for enrollment. A total of 190 consecutive CLMs from 125 consecutive patients who met the criteria were collected. Three ablation modalities were used on the study population: microwave, radiofrequency, or irreversible electroporation. 

## 3. Study Method

A non-contrast CT and first FDG injection PET acquisition were obtained in each case to localize the CLM immediately prior to ablation. The ablation was performed with the intent to create an AZ at least 10 mm larger than the largest tumor diameter to achieve a minimal margin (MM) of at least 5 mm all around the targeted CLM. The study’s aim is to evaluate whether the addition of PET to CT increases tumor detection prior to ablation and definition of tumor contour for AZ and margin assessments.

### PET and CT-Acquisition Protocols

PET scans were centered in the liver and obtained for one or two bed positions consisting of 47 transverse slices, 3.27 mm thick, with 11 slice overlap. A transaxial field of view of 70 cm and 128 × 128 image matrix (5.47 × 5.47 mm pixels) were used for all cases. The acquisition time typically varied from 2 to 5 min per bed for the tumor localization PET scan. The fusion of the attenuation correction CT with PET was always inspected and used for tumor identification and lesion segmentation. Additional CT scans were obtained after electrode placement and fused with the pre-ablation PET. Additional real-time PET/CT acquisitions after electrode placement in the targeted CLM were also obtained as needed using a 2 min acquisition time to depict the electrode(s) in relation to the targeted CLM. One-minute breath-hold (apnea) acquisitions were also obtained as needed for tumors in subphrenic positions to eliminate misregistration artifacts due to diaphragmatic/respiration motion.

## 4. Split-Dose PET

This method has been previously described in detail [20]. In short, the split-dose PET method uses the same total FDG dose as a diagnostic PET (12 mCi (444 MBq)) divided into two aliquots. The first aliquot represents one-third of the diagnostic dose (4 mCi (148 MBq) and is used as the initial tumor-targeting dose, administered within 60 min of ablation. The second aliquot treatment efficacy is dose-equivalent to two-thirds of the diagnostic dose (8 mCi (296 MBq) and is administered upon completion of the ablation to assess the treated tumor and the AZ. The 4-mCi (148-MBq) dose produces enough signal to depict the targeted tumor for localization and targeting. The fusion of this low-dose PET signal with the CT aids in accurate tumor identification and localization for targeting as well as subsequent imaging and monitoring during the ablation without the need for additional FDG or contrast injections. The technique of sequential, differential dosing has been validated in multiple studies [21,22].

## 5. Image Analysis

**Figure 2:**
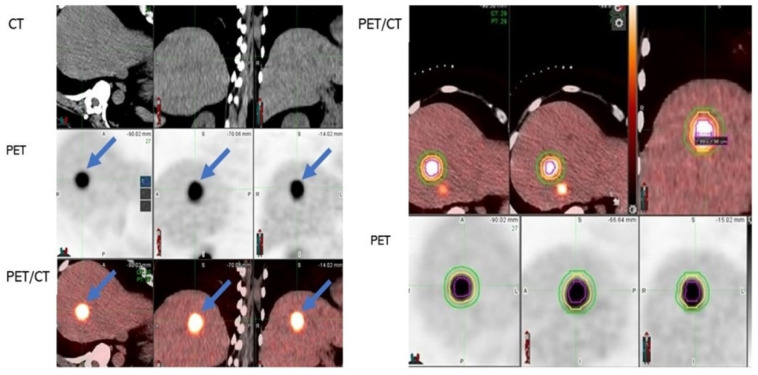
CLM detection and margin assessment on pre-ablation imaging.

**Figure 3:**
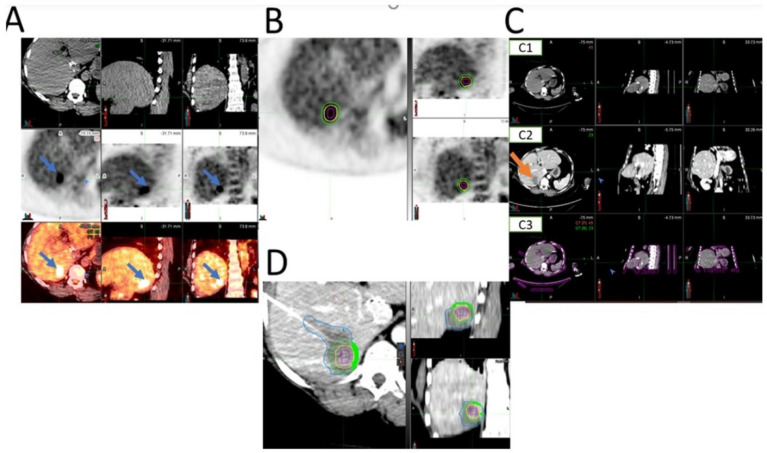
Steps in assessment of intraprocedural PET/CT guided ablation. (**A**) Accurate detection of tumor on pre-ablation imaging modalities is the first step (here, the tumor was detectable on PET and PET/CT—shown with blue arrows—but not on CT alone). (**B**) Segmentation of tumor (red circle) and margins (5−mm margin depicted with yellow circle and 10−mm depicted with green circle) on pre-ablation PET. (**C**) Registration of pre-ablation non-contrast CT (C1) and post-ablation portal venous CT (C2) (registration is shown in C3). The ablation zone is shown with an orange arrow on the portal venous phase of the post-ablation contrast-enhanced CT (C2). (**D**) The tumor (red circle), 5−mm (yellow circle), and 10−mm (green circle) margins transferred from pre-ablation PET to the registered post-ablation portal venous CT for assessment of the tumor and tumor margins that are not covered within ablation zone AZ (AZ is depicted with blue here; the margins that are not covered by the AZ are filled with green).

Non-contrast diagnostic CT and PET/CT images before ablation were visually assessed for the detection of the targeted CLM. Digital imaging and communications in medicine (DICOM) files were opened with MIM software (version 6.9.7, Cleveland, OH, USA) (Figure 2). The liver window in the MIM software was chosen for assessment of the CLM on CT. *FDG* avidity greater than the physiologic liver background matching the tumor location and size was interpreted positively on the pre-ablation PET/CT (Figure 3). CLMs were divided into detectable, non-detectable, and poor conspicuity on CT and detectable, non-detectable, and low FDG avidity on PET/CT, based on how accurately they could be characterized on imaging modalities (Figure 3). In order to perform the 3D volumetric evaluation, the tumor must be initially detected on the intraprocedural pre-ablation images (Figure 2). Then, the tumor contour and the expected 5- and 10-mm margins are contoured on the PET scan. The imaging contours are then transferred from the pre-ablation imaging to the registered non-contrast CT and the post-ablation portal venous phase, used for the assessment of the AZ and the detection of areas that are insufficiently covering the targeted tumor by the AZ (Figure 2 and Figure 3). Contrast-enhanced CT was always obtained upon completion of the TA for evaluation of the AZ, as previously described [8].

A 49-year-old woman with recurrent adenocarcinoma of the colon with recent evidence of liver metastasis underwent microwave ablation. The image set on the left shows a CLM detected on PET but with poor conspicuity on CT alone. Because of the unclear depiction of the tumor contour, the 5− and 10−mm margins in this patient could not be assessed using CT alone. In the images on the right, a gradient-based algorithm was used to segment the tumor. The 5− and 10−mm tumor margins are shown with yellow and green contours in the PET and fused PET/CT images (*MIM software*, *version* 6.9.7, Cleveland, OH, USA).

## 6. Statistics

Nominal data are presented as frequency and percent. Phi and Cramer’s V chi-square-based were used to measure the association between the nominal data. Pie charts represent the percentage of the observations of a level for each imaging modality. The sample size was determined to allow 90% power to assess the value of the added PET. The level of significance was set at *p* < 0.05. IBM SPSS Statistics 26.0 was used for data analysis (released 2019. IBM SPSS Statistics for Windows, Version 26.0. Armonk, NY, USA: IBM Corp). The study statistician (MG) confirmed the statistics in this study. He has been at MSKCC since 1999. He focuses on the epidemiology and statistics of colorectal metastatic disease and has been serving as Chief of the Biostatistics Service since 2015.

## 7. Results

**Figure 4:**
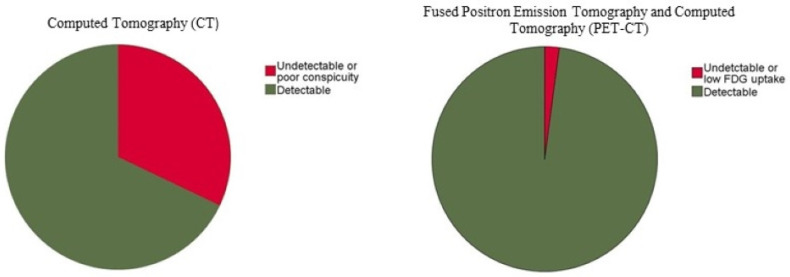
Pie charts showing the colorectal liver metastasis detection rate for CT and PET/CT.

**Figure 5:**
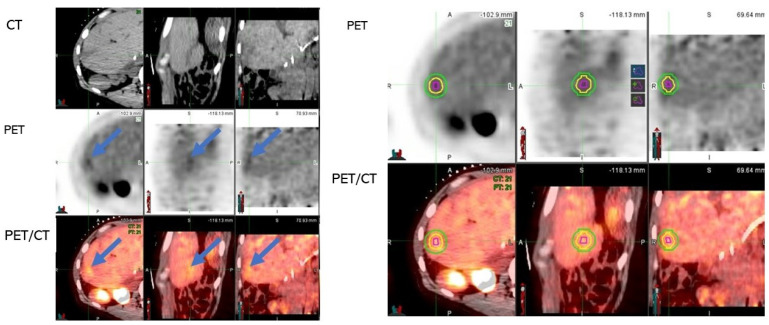
An example of a CLM with low-FDG avidity and 5− and 10−mm margins assessment for the target CLM.

**Figure 6:**
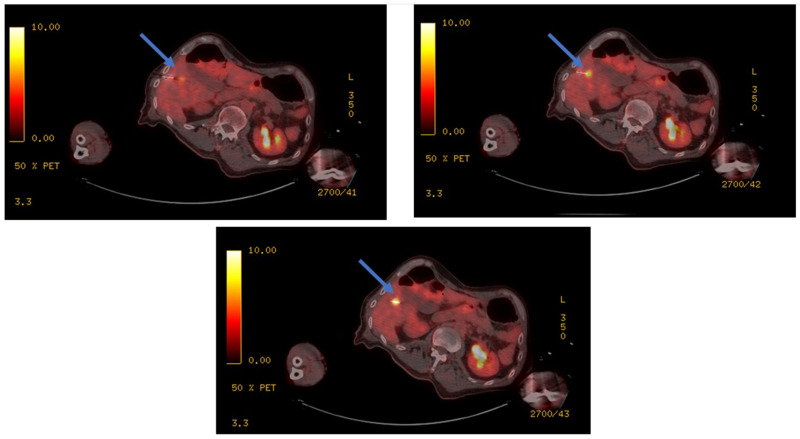
Real-time PET provides the benefit of continuous depiction of the FDG avid CLMs during the ablation and eliminates the need for multiple contrast injections. The location of the tumor and the ablation electrode is depicted with blue arrows in three consecutive PET/CT slices.

A total of 190 CLMs underwent thermal ablation in 125 patients (median age: 56.24 years; interquartile range: 44.54–64.89) using the real-time split-dose PET/CT guidance method analyzed. A total of 129/190 (67.9%) CLMs were detectable on non-contrast CT (CT) alone, while 61 CLMs (32.1%) were either undetectable or could not be properly defined on CT due to insufficient conspicuity (lesion detection rate: 67.9%) (Table 2 and Figure 4). Because of the imprecise portrayal of the tumor contour and edges, the theoretical 5- and 10-mm margins could not be defined in these CLMs (32.1%) (Figure 3 and Figure 5). Only 4 CLMs (2.1%) remained undetectable or had a low FDG avidity (Figure 6) after fusing the pre-ablation PET signal with the pre-ablation CT (fused PET/CT). Most CLM were evident on PET/CT (CLM detection rate: 97.9%) (Table 2 and Figure 4). CT and PET/CT had a 69% concordance for detecting CLM. In Phi and Cramer’s V analyses, there was a moderate association between the two imaging modalities (co-efficient: 0.135, approximate significance: 0.063) (Table 3).

The red portions of the pie charts represent undetectable or poor lesion detection on imaging modalities, which decreased from 32.1% to 4% after integrating PET and CT scans (fused PET/CT).

## 8. Discussion

This study demonstrated that the combination of real-time FDG-PET with pre-ablation non-contrast-enhanced CT significantly improved CLM detection and contour depiction and eliminated the need for contrast administration prior to and during TA for targeting the CLM. Only four CLMs (2.1%) remained undetectable or had a very low FDG avidity. Even in the lesions with low FDG uptake, the margins could be assessed using a 3D volumetric approach (Figure 5). Real-time PET also allows continuous tumor depiction during ablation (Figure 6). After the initial injection of one-third of the diagnostic FDG dose, real 2 min PET acquisitions can be obtained as needed to depict the targeted FDG avid tumor (Figure 6).

A 46-year-old female participant with a history of hepatic metastasis from colorectal adenocarcinoma underwent microwave ablation. The tumor was not KRAS mutated, but P53 was positive. The patient had chemotherapy through a hepatic artery infusion before the ablation. Low FDG uptake was observed and quantified for the CLM (maximum standardized uptake value (SUVmax): 2.98, total lesion glycolysis: 0.7, metabolic tumor volume: 0.24). Low FDG uptake is due to various reasons, including low glucose metabolism, low cellularity or small tumor size, recent chemotherapy, and absence of KRAS mutations. The CLM was not evident on pre-ablation non-contrast CT. The right imaging set shows that despite low FDG uptake, the tumor was still segmented on PET and PET/CT using a gradient-based algorithm. The 5- and 10-mm margins were also assessed based on the tumor boundaries (MIM software, version 6.9.7, Cleveland, OH, USA).

**Figure 7:**
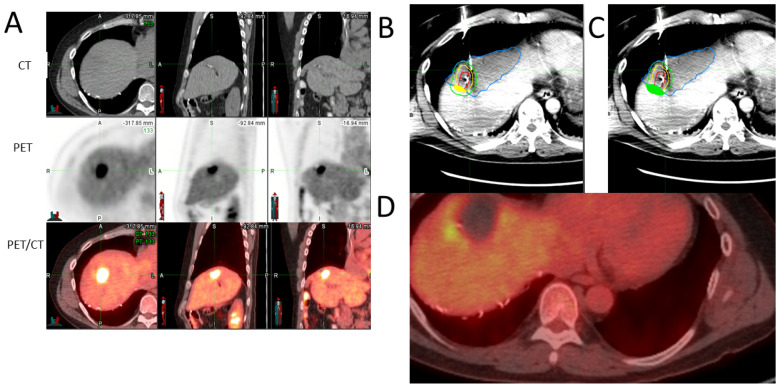
Importance of accurate tumor and margins detection for preventing local tumor progression. A patient with colorectal liver metastasis in segment8. (**A**) Pre-ablation anatomical imaging (CT) and PET and fused PET/CT. The CLM was detected on PET and PET/CT but not easily detectable on CT alone. Insufficient coverage of margins (5 mm margin filled with yellow (**B**) and 10 mm margin filled with green (**C**)) led to tumor progression (**D**) in less than 6 months after thermal ablation in the area where the tumor was not sufficiently covered.

LTP remains an important limitation of tumor ablation. LTP is often the result of insufficient stereotactic coverage of the targeted tumor by the AZ with sufficient ablation margins [6,7,8,9,10,11,12,13,14,15,17] (Figure 7). Therefore, the accurate detection of CLM for precise targeting, monitoring, and subsequent assessments of the AZ and calculation of margins are critical for successful local tumor control using ablation [12]. Our group previously demonstrated that the combination of FDG-PET with immediate mandatory post-ablation CECT improved the ability to predict 12-month LTP after TA [22].

The high detection rate of CLM with PET observed in this study is in accordance with the results of previous studies [23,24,25]. The result of a metanalysis showed that FDG PET had a higher detection rate for liver metastasis in comparison to helical CT with contrast and MRI [24]. The results demonstrated that the sensitivities for detection of liver metastasis were 60.2% for nonhelical CT (*n* = 1915), 64.7% for helical CT with contrast (*n* = 621), 75.8% for 1.5-T MRI (*n* = 391), and 94.6% for FDG PET (*n* = 1058) [24].

Accurate CLM detection for targeting, monitoring, and subsequent identification of the tumor areas not covered by the AZ with adequate minimal margins (MM under 5 mm) is crucial to achieving local tumor control (Figure 7). LTP rates of up to 48% have been reported after ablation, limiting the widespread use of this therapy for a local cure for CLM [26]. Tumor size, proximity to vessels, and in particular, MM are well-known factors associated with local tumor control [6,27,28]. An adequate MM covering the entire targeted CLM is considered the most important technical factor for local tumor control (Figure 7). A MM over 5 mm, and ideally over 10 mm, in all directions around the target, is associated with local tumor control [6,21,29,30]. LTP rates of 5 and 15% have been reported for CLM ablated with 5–10 and >10 mm MM, respectively, within a 55-month follow-up period [6]. A prospective single-arm trial adding biopsy of the AZ immediately after CLM RFA achieved 97% local progression-free survival at 30 months for CLM ablated with tumor-negative biopsy and MM > 5 mm [30]. A recent, larger study validated these findings for both RFA and microwave ablation with 93% local tumor control at 12 months for biopsy-negative AZ with MM > 5 mm [31]. For biopsy-proven tumor-negative AZs, the MM size and SUV ratio were predictive surrogate imaging biomarkers of LTP [21]. Volumetric assessment of the MM has been used as an intraprocedural tool for ablation success [15] and a MM of 5 mm in a PET/CT ablation series correlated well with LTP [32]. In the current study, because of the imprecise portrayal of the tumor contour and edges, the theoretical 5- and 10-mm margins could not be defined in 32.1% of CLMs using CT alone. A series of ammonia perfusion PET during FDG PET/CT-guided liver tumor ablation showed that no tumors ablated with a MM > 0 mm progressed locally [33]. In mutant CLM, broader MM is needed to achieve local tumor control [9]. Specifically, a MM of >5 mm is absolutely required for meaningful local tumor control [9]. While no LTP was documented for CLM ablated with MM > 10 mm [11,13], a dedicated study recorded a higher LTP rate for KRAS-mutant CLM even when ablated with MM > 10 mm [9].

Specifically, this study documented a moderate association between CT and PET/CT for CLM detection pre-ablation. Considering that 98% of CLMs were detected on PET/CT but only 62.9% on non-CECT, the added value of PET, when combined with CT to detect, monitor, and segment the targeted CLM for ablation and the subsequent assessment of the AZ and margin is clear.

This study has limitations. The split-dose method requires the involvement of the nuclear medicine staff for FDG injections and operating the PET scanner during ablation. The limited number of PET/CT scanners worldwide and the even smaller number of dedicated PET/CT equipment allowing the presence of anesthesia equipment and staff for image-guided interventions currently impact the utility of this method in most institutions [21]. Although 3D software is increasingly used and is recommended for the proper assessments of the AZ [8,34,35], its widespread availability is still lacking. All software platforms are affected by motion artifacts and extreme positioning differences that impact registration and fusion. Real-time PET/CT can help overcome these issues, especially with the option to acquire additional PET/CT after electrode placement or protective maneuvers such as hydrodissection. Despite the limitations, the study showed the value of intraprocedural PET imaging for tumor ablation, allowing for improved tumor detection for ablation targeting, monitoring, and segmentation for post-ablation assessments. This and similar work encourage and justify the needed of investment for the evolution of PET/CT and 3D software platforms use in image-guided tumor ablation [21].

## 9. Conclusions

The described integration of intraoperative FDG/PET to CT improved targeted tumor depiction, the mandatory first critical step for accurate ablation targeting, monitoring, and segmentation for margin assessment. These steps are critical components for local tumor control and optimal oncologic outcomes after CLM ablation and can be used for TA of any FDG-avid liver tumor.

## Figures and Tables

**Table 1:** Participant/Lesion Characteristics.

Number of Participants/CLMs: 125/190
Age * Median: 56.24 years (Interquartile range: 44.54–64.89)
Primary Diagnosis Colon: 180 (94.7%) Rectal: 9 (4.7%) Appendix: 1 (0.5%)
Histology Adenocarcinoma not otherwise specified: 1 (0.5%) Moderately differentiated adenocarcinoma:168 (88.4%) Moderately to poorly differentiated adenocarcinoma:3 (1.6%) Poorly differentiated adenocarcinoma: 15 (7.9%) Well-differentiated adenocarcinoma: 2 (1.1) Unknown: 1 (0.5%)
Akt1 Mutation Lesion testing unavailable: 86 (45.3%) No: 101 (53.2%) Pending: 2 (1.1%) Yes: 1 (0.5%)
BRAF mutation ** Lesion testing unavailable: 86 (45.3%) No: 99 (52.1%) Pending: 2 (1.1%) Yes: 3 (1.6%)
EGFR mutation ** Lesion testing unavailable: 86 (45.3%) No: 98 (51.6%) Pending: 2 (1.1%) Yes: 4 (2.1%)
KRAS mutation** Lesion testing unavailable: 86 (45.3%) No: 60 (31.6%) Pending: 2 (1.1%) Yes: 42 (22.1%)
PIK3CA mutation ** Lesion testing unavailable: 86 (45.3%) No: 90 (47.4%) Pending: 2 (1.1%) Yes: 12 (6.3%)

* Age is represented for participant characteristics. The rest are lesion characteristics. ** BRAF (v-raf murine sarcoma viral oncogene homolog B1), EGFR (epidermal growth factor receptor), KRAS mutation: Kirsten rat sarcoma viral oncogene homolog, PIK3CA: Phosphatidylinositol-4,5-Bisphosphate 3-Kinase Catalytic Subunit Alpha.

**Table 2:** Lesion detection rate for CT and PET/CT.

		Frequency	Percent
CT	Non-detectable or poor conspicuity	61	32.1
	Detectable	129	67.9
	Total	190	100.0
PET/CT	Non-detectable or low FDG avidity	4	2.1
	Detectable	186	97.9
	Total	190	100.0

**Table 3:** Phi and Cramer’s V chi-square-based measures of nominal association between two imaging modalities.

Symmetric Measures			
		Value	Approximate Significance
Nominal by Nominal	Phi	0.135	0.063
	Cramer’s V	0.135	0.063
N of Valid Cases		190	

## Data Availability

Data will be available from the corresponding author upon reasonable requests.

## Abbreviation

AZ = ablation zone, BRAF: v-raf murine sarcoma viral oncogene homolog B1, CECT = contrast-enhanced CT, CLM = colorectal cancer liver metastasis, CT: computed tomography, CRC = colorectal cancer, CT = computed tomography, DICOM = digital imaging and communications in medicine, EGFR: epidermal growth factor receptor, FDG = fluorodeoxyglucose, HIPPA: Health Insurance Portability and Accountability Act of 1996, IRB: institutional review board, KRAS = mutant Kristen rat sarcoma viral oncogene homolog, LTP = local tumor progression, LTPFS: liver tumor progression-free survival, MBq: megabecquerel, mCi: millicurie, MM = minimum ablation margin, *, MRI: magnetic resonance imaging, NCI/NIH = *National Cancer Institute*/National Institute of Health, PET: positron emission tomography, PIK3CA: phosphatidylinositol-4,5-bisphosphate 3-kinase catalytic subunit alpha, SUVmax: maximum standardized uptake value, TA = thermal ablation
